# *In silico* screening of known small molecules to bind ACE2 specific RBD on Spike glycoprotein of SARS-CoV-2 for repurposing against COVID-19

**DOI:** 10.12688/f1000research.24143.1

**Published:** 2020-07-01

**Authors:** Bharath BR, Hrishikesh Damle, Shiban Ganju, Latha Damle

**Affiliations:** 1 Computational Biology, Atrimed Biotech LLP, Banglore, 560100, India; 2 Atrimed Pharmaceuticals Pvt. Ltd, Banglore, 560001, India

**Keywords:** SARS-CoV-2, Pathogen host interaction, SARS-CoV-2-Spike Glycoprotein, ACE2, Small Molecules

## Abstract

**Background**: Human coronavirus (SARS-CoV-2) is causing a pandemic with significant morbidity and mortality. As no effective novel drugs are available currently, drug repurposing is an alternative intervention strategy. Here we present an *in silico* drug repurposing study that implements successful concepts of computer-aided drug design (CADD) technology for repurposing known drugs to interfere with viral cellular entry via the spike glycoprotein (SARS-CoV-2-S), which mediates host cell entry via the hACE2 receptor.

**Methods**: A total of 4015 known and approved small molecules were screened for interaction with SARS-CoV-2-S through docking studies and 15 lead molecules were shortlisted. Additionally, streptomycin, ciprofloxacin, and glycyrrhizic acid (GA) were selected based on their reported anti-viral activity, safety, availability and affordability. The 18 molecules were subjected to molecular dynamics (MD) simulation.

**Results**: The MD simulation results indicate that GA of plant origin may be repurposed for SARS-CoV-2 intervention, pending further studies.

**Conclusions**: Repurposing is a beneficial strategy for treating COVID-19 with existing drugs. It is aimed at using docking studies to screen molecules for clinical application and investigating their efficacy in inhibiting SARS-CoV-2-S. SARS-CoV-2-S is a key pathogenic protein that mediates pathogen-host interaction. Hence, the molecules screened for inhibitory properties against SARS-CoV-2-S can be clinically used to treat COVID-19 since the safety profile is already known.

## Introduction

The complete genome of severe acute respiratory syndrome coronavirus 2 (SARS-CoV-2) is 82% identical to SARS-CoV, both viruses share a common clade encompassing the genus *Betacoronavirus* as the root node ^1, 2^. Currently no novel antivirals exist that are effective against either of the viruses ^3– 6^.

Drug repurposing is a commercially viable strategy, as it exploits existing drugs, thus significantly reducing the cost and time involved in developing effective therapeutics ^7– 9^. Experimental approaches, however, at pre-clinical and clinical stages for drug repurposing involve high cost and time ^10^. Computational approaches can offer quick, considerable, and novel testable hypotheses for systematic drug repositioning ^8^.

Current drugs in different phases of clinical trials are being investigated for inhibitory activity against viral targets that play a significant role in the coronavirus infection lifecycle. The drug targets might be involved in entry into the host (e.g. umifenovir and chloroquine), replication (e.g. lopinavir/ritonavir), or RNA synthesis (e.g. remdesivir/favipiravir). Among these, targeting SARS-CoV-2 cellular entry via the spike glycoprotein (SARS-CoV-2-S) has emerged as the leading option for repurposing ^9^. As SARS-CoV-2-S is a surface protein involved in adhesion/fusion and entry into host cells, it has been identified as a potential drug target for both biologics and small molecules ^10^.

The entry of COVID-19 pathogen is mediated by the homotrimeric transmembrane protein SARS-CoV-2-S. It is comprised of two functional subunits, S1 and S2, which are non-covalently bound in the pre-fusion conformation. The S1 subunit interacts with the human ACE2 receptor through the receptor binding domain (RBD), while the S2 subunit is one of the components of viral envelope ^11– 19^.

Apart from interacting with the ACE2 receptor, the RBD site also contributes to the stabilisation of the prefusion state of the S2 subunit equipped with fusion machinery ^18– 24^. In CoVs, the S-protein is cleaved by host proteases at the S20 site located above the fusion peptide ^16, 25^. This activates the protein via extensive irreversible conformational changes ^11, 16, 17, 23, 26^. It is well understood that the entry of CoV into the susceptible host is a complex process that requires the vigorous actions of receptor binding and proteolytic processing of the S-protein to promote fusion with the pathogen ^27^.

Hence, the current study aims to predict and validate the structure of SARS-CoV-2-S protein using computer-aided homology modelling tools and screen a library of small molecules for their interaction with the SARS-CoV-2-S protein.

## Methods

### Sequence analysis

The whole genome of SARS-CoV-2 (GenBank accession number: MT159721.1, length: 29882 bp) was retrieved from NCBI and used as a query to perform a sequence similarity search using NCBI-BLAST ^28^. The BLAST search revealed 96.04%, 91.64% and 82.30% identity with Bat coronavirus RaTG13 (GenBank accession number: MN996532.1, length: 29855 bp), Pangolin coronavirus isolate MP789 (GenBank accession number: MT084071.1, length: 27213 bp) and SARS-CoV (GenBank accession number: JX163927, length: 29646bp), respectively.

### Topological analysis of pathogen-host interactome for target validation

Drug target identification and validation were carried out using a network-based topological analysis method using the web-based application Pathogen-Host Interaction Search Tool (PHISTO) by setting pathogen type to virus, family to coronaviridae, species to SARS-Related coronavirus and strain to SARS-Cov. The node properties like the degree of connectivity (k) and betweenness centrality (BC) were assessed ^29, 30^. The statistical significance of k and BC values were assessed by the Fligner-Killeen (median) test.

### Prediction of ligand binding site

The similarity between RBD domains of S-protein from SARS-CoV-2 (accession number QII57328, length:1273aa), SARS-CoV (accession number: AFR58728, length:1255aa) and RatG13 (accession number: QHR63300, length:1269aa) was evaluated by using the multiple sequence alignment (MSA) tool Clustal Omega from EMBL-EBI. Conservation in ACE2 receptor interaction was seen among all the three sequences aligned. This conservation aided in the active binding site prediction.

The protein-protein interaction between SARS-CoV-2-S and host ACE2 receptor complex was studied using the crystal structure from Protein Data Bank (PDB ID: 6CS2). The amino acids involved in the interaction were identified as ligand binding sites for inhibitor molecules.

### Homology modelling of SARS-CoV-2-S protein

Homology modelling was performed with SWISS-MODEL for the protein sequence of SARS-CoV-2-S using the crystal structure of SARS-CoV-S and ACE2 complex (PDB ID: 6ACD) as a template. The modelled protein was validated for quality using a Ramachandran plot and prepared for molecular docking studies using the Protein Preparation Wizard feature of the Schrodinger Small Molecule Suite ^31^. This analysis could also have been performed using open source software such as AutoDock ^32^ or SwissDock ^33^.

### Protein preparation

The modelled receptor was processed for docking studies by deleting crystallographic water molecules with less than three H-bonds. This could also be done manually by editing the .PDB file in a text editor. Next, hydrogen atoms corresponding to neutral pH were added in consideration of ionisation states of amino acids. Following this, coordinates for any missing side-chain atoms were added using Prime v4.0, Schrödinger 2019-2 ^34^. Finally, the energy of the modelled structure was minimised using the OPLS_2005 force field ^35^. This analysis could also have been performed using open source software such as AutoDock ^32^.

### Ligand preparation

The three-dimensional conformations of the 4015 small molecule drugs already in use to treat various diseases and as nutritional supplements were downloaded from the DrugCentral database and subjected to ligand minimisation using Ligprip (LigPrep, version 2.3, Schrödinger, LLC, New York, NY, 2009). This analysis could also have been performed using open source software such as AutoDock ^32^. The compounds were minimised by assigning force field OPLS_2005 and stereoisomers were calculated after retaining specific chiralities. The absorption, distribution, metabolism and excretion (ADME) predictions were performed for all ligands using the QikProp package ^36^. This analysis could also have been performed using open source software such as SWISS-ADME ^37^.

### Molecular docking

The active site on the prepared receptor was defined around the selected residues (Arg426, Tyr436, Pro462, Thr486, Gly488, and Tyr491) with a 10Å radius. This generated a grid box measuring 20X20X20Å. The docking of small molecules over SARS-CoV-2-S was performed using Glide v7.8, This analysis could also have been performed using open source software such as AutoDock ^32^. Schrödinger 2019-2 ^38^ in different modes sequentially with defined and incremental precision, and computational time differences. The best-docked conformer with minimum Glide energy and E model energy was selected and lowest-energy docked complex of three known molecules streptomycin, ciprofloxacin, and glycyrrhizic acid (GA) in complex with SARS-CoV-2-S were selected for molecular dynamic simulations.

### Molecular dynamics (MD) simulations

The MD ^39^ of shortlisted complexes were studied using the OPLS_2005 force field ^40^ in a plane TIP3P water model ^41^. MD simulations were performed using Desmond version 4.2 ^42^. This analysis could also have been performed using open source software such as GROMACS ^43^. The system was built by dissolving the streptomycin/SARS-CoV-2-S, ciprofloxacin/SARS-CoV-2-S, and GA/SARS-CoV-2-S complexes in an orthorhombic box containing water molecules, allowing a buffer region of 10Å between atoms and box peripherals. The system was further minimised using the L-BFGS algorithm for a minimum of 10 steepest descent steps and a maximum of 2000 iterations until a gradient threshold of 25 kcal/mol/Å and convergence threshold of 1.0 kcal/mol/Å was reached. For short-range electrostatic interactions, the solid-phase microextraction ^44^ method was employed at 1e-09 tolerance and 9Å cut-off radius. The built systems were gradually warmed up to 300K in the NPT ensemble with a time step of 2fs. A 100ns MD simulation in the NPT ensemble was performed using a Nose–Hoover thermostat ^40^. Resulting root mean square deviation (RMSD) and root mean square fluctuation (RMSF) values were analysed.

## Results and discussion

### Multiple sequence alignment of complete genomes

**Figure 1.**
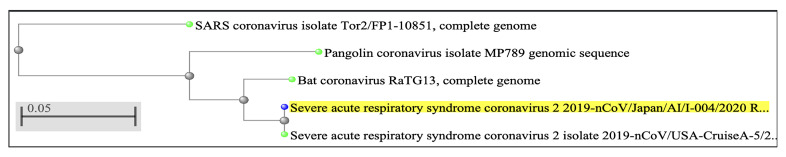
Cladogram constructed using neighbour joining tree construction method, depicting the distance between the complete genome sequences subjected for multiple sequence alignment. This figure was generated using EMBL-EBI Clustal Omega.

The complete genome of coronavirus SARS-CoV-2, Bat coronavirus RaTG13, Pangolin coronavirus isolate MP789, and SARS-CoV obtained as BLAST hits were aligned and a phylogenetic tree was constructed ( Figure 1).

The MSA demonstrated the molecular similarities between the organisms. RaTG13 has been identified as a neighbour genome for SARS-CoV-2 and this justifies the hypothesis that the infection may be transmitted from bats. Meanwhile, the subsequent neighbours were Pangolin MP789 and SARS-CoV. This preliminary sequence alignment enabled the understanding of sequence similarities and evolutionary information, which is deeply fundamental to the process of drug discovery.

### Topological analysis and target validation

**Figure 2.**
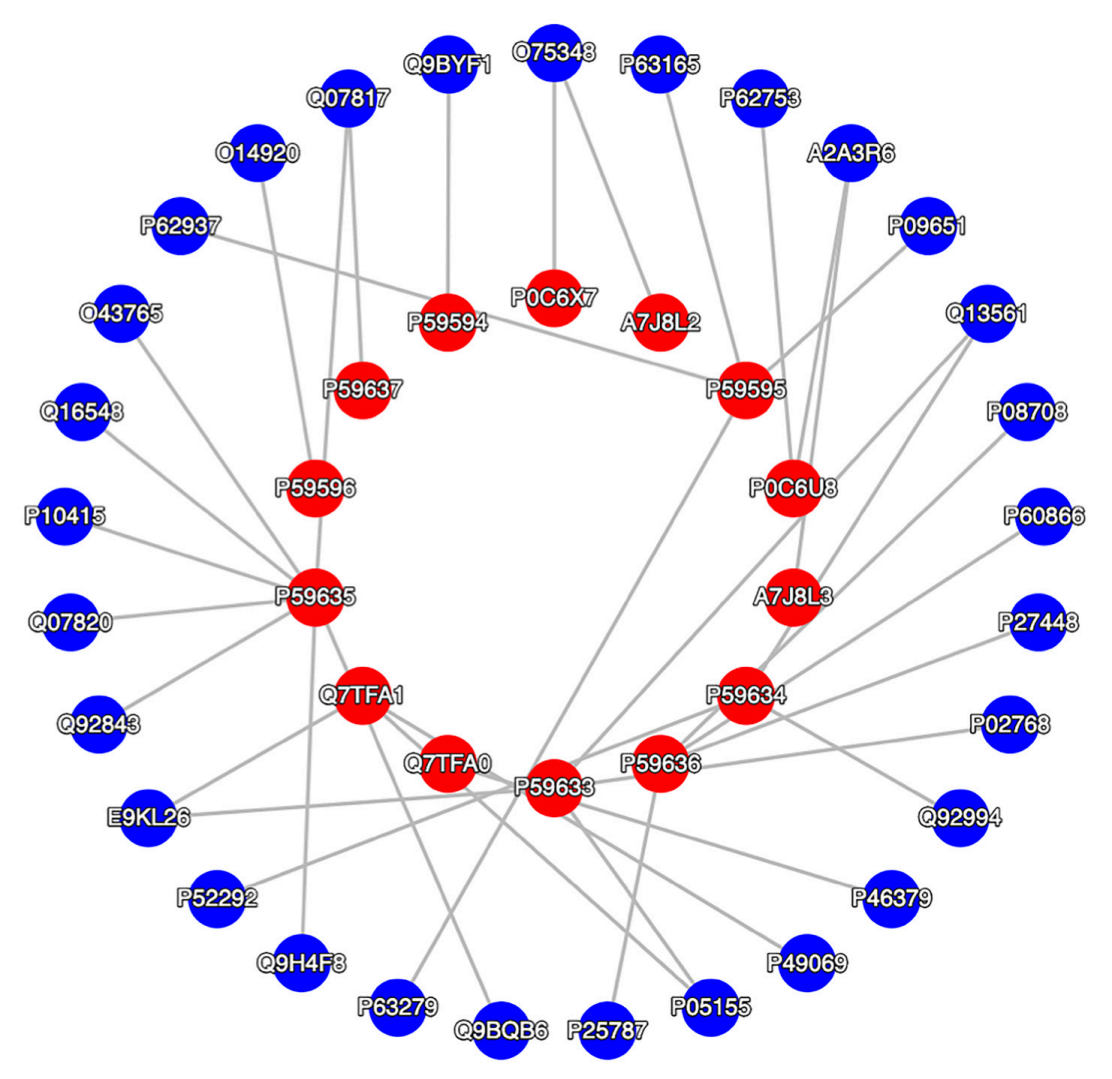
The SARS-CoV/human pathogen-host interactome illustrating the protein-protein interactions between proteins. The 28 blue nodes correspond to human proteins and 14 red nodes are SARS-CoV-2 proteins. The nodes are connected by 35 edges. This figure was generated using Pathogen-Host Interaction Search Tool (PHISTO).

A detailed investigation of the pathogen-host interactome can shed clear insights on the mechanism of viral infection and the pathology involved. Due to a lack of interaction data on SARS-CoV-2, the SARS-CoV proteome was considered and the SARS-CoV/human interactome was built by screening domain interactions between SARS-CoV/human protein-protein interactions, and then the network distribution, topological and functional analyses were performed ( Figure 2). The circular shapes correspond to proteins (nodes) which are labelled by Uniprot_IDs and details about the nodes are listed in Table 1.

Among 14 proteins of SARS-CoV, the majority of SARS-CoV/human interaction involves five non-structural proteins (NS3B, NS6, NS7A, NS7B and NS8A with four, three, eight, three and one human proteins, respectively), three open reading frame (ORF) polyproteins (ORF9B, A7J8L3 and A7J8L2 with four, one and one human proteins, respectively), two replicase proteins (R1A and R1AB with two and one human proteins, respectively), the Membrane protein (VME1 with human IKKB), Envelope membrane protein (VEMP with human B2CL1), Nucleoprotein (NCAP with four human proteins) and Spike glycoprotein (SPIKE with human ACE2). With these observations, we determine the high specificity of Membrane, Envelope and Spike glycoprotein interactions with the host through specific entry points. Hence, these three SARS-CoV proteins can be a potential target to inhibit the pathogen-host interaction specifically, while other interactions are more versatile. To ensure the impact of inhibition of IKKB, B2CL1, and ACE2 mediated interaction, the landscape of the SARS-CoV/human interaction was further analysed for degree and betweenness centrality distributions of the host, as shown in Table 2.

**Table 1.** The list of proteins involved in SARS-CoV/human interactions with experimental methods and Pubmed ID as reference.

Uniprot ID	Pathogen protein	Uniprot ID	Human protein	Experimental method	Pubmed ID
P59596	VME1	O14920	IKKB	Coimmunoprecipitation	17705188
P59637	VEMP	Q07817	B2CL1	Coimmunoprecipitation	16048439
P59637	VEMP	Q07817	B2CL1	Other Methods	16048439
P59594	SPIKE	Q9BYF1	ACE2	Experimental interaction detection	18343844
P59594	SPIKE	Q9BYF1	ACE2	Pull down	23486063
P0C6U8	R1A	P62753	RS6	Anti tag coimmunoprecipitation	19838190
P0C6U8	R1A	A2A3R6	A2A3R6	Anti tag coimmunoprecipitation	19838190
P0C6X7	R1AB	O75348	VATG1	Anti bait coimmunoprecipitation	16226257
P0C6X7	R1AB	O75348	VATG1	Anti tag coimmunoprecipitation	16226257
P0C6X7	R1AB	O75348	VATG1	Experimental interaction detection	16226257
P0C6X7	R1AB	O75348	VATG1	Phage display	16226257
P0C6X7	R1AB	O75348	VATG1	ELISA	16226257
P59636	ORF9B	P08708	RS17	Two hybrid	22046132
P59636	ORF9B	P60866	RS20	Two hybrid	22046132
P59636	ORF9B	P27448	MARK3	Two hybrid	22046132
P59636	ORF9B	P25787	PSA2	Two hybrid	22046132
P59636	ORF9B	P25787	PSA2	LUMIER	22046132
P59636	ORF9B	P27448	MARK3	LUMIER	22046132
P59636	ORF9B	P60866	RS20	LUMIER	22046132
P59636	ORF9B	P08708	RS17	LUMIER	22046132
Q7TFA0	NS8A	P46379	BAG6	Two hybrid	22046132
Q7TFA0	NS8A	P46379	BAG6	LUMIER	22046132
Q7TFA1	NS7B	P49069	CAMLG	Two hybrid	22046132
Q7TFA1	NS7B	P05155	IC1	Two hybrid	22046132
Q7TFA1	NS7B	E9KL26	E9KL26	Two hybrid	22046132
Q7TFA1	NS7B	P05155	IC1	LUMIER	22046132
Q7TFA1	NS7B	P49069	CAMLG	LUMIER	22046132
Q7TFA1	NS7B	E9KL26	E9KL26	LUMIER	22046132
P59635	NS7A	Q9BQB6	VKOR1	Two hybrid	22046132
P59635	NS7A	Q9H4F8	SMOC1	Two hybrid	22046132
P59635	NS7A	Q92843	B2CL2	Coimmunoprecipitation	17428862
P59635	NS7A	Q07820	MCL1	Coimmunoprecipitation	17428862
P59635	NS7A	P10415	BCL2	Coimmunoprecipitation	17428862
P59635	NS7A	Q16548	B2LA1	Coimmunoprecipitation	17428862
P59635	NS7A	O43765	SGTA	Coimmunoprecipitation	16580632
P59635	NS7A	Q07817	B2CL1	Coimmunoprecipitation	17428862
P59635	NS7A	Q9BQB6	VKOR1	LUMIER	22046132
P59635	NS7A	Q9H4F8	SMOC1	LUMIER	22046132
P59635	NS7A	O43765	SGTA	Other Methods	16580632
P59634	NS6	Q13561	DCTN2	Two hybrid	22046132
P59634	NS6	Q92994	TF3B	Two hybrid	22046132
P59634	NS6	P52292	IMA1	Two hybrid	17596301
P59634	NS6	Q13561	DCTN2	LUMIER	22046132
P59634	NS6	Q92994	TF3B	LUMIER	22046132
P59633	NS3B	Q13561	DCTN2	Two hybrid	22046132
P59633	NS3B	P02768	ALBU	Two hybrid	22046132
P59633	NS3B	P05155	IC1	Two hybrid	22046132
P59633	NS3B	E9KL26	E9KL26	Two hybrid	22046132
P59633	NS3B	P05155	IC1	LUMIER	22046132
P59633	NS3B	Q13561	DCTN2	LUMIER	22046132
P59633	NS3B	P02768	ALBU	LUMIER	22046132
P59633	NS3B	E9KL26	E9KL26	LUMIER	22046132
P59595	NCAP	P63165	SUMO1	Anti tag coimmunoprecipitation	15848177
P59595	NCAP	P09651	ROA1	Two hybrid	15862300
P59595	NCAP	P63279	UBC9	Two hybrid	16998888
P59595	NCAP	P63279	UBC9	Coimmunoprecipitation	16998888
P59595	NCAP	P63165	SUMO1	Coimmunoprecipitation	15848177
P59595	NCAP	P62937	PPIA	Coimmunoprecipitation	15688292
P59595	NCAP	P09651	ROA1	Pull down	15862300
P59595	NCAP	P09651	ROA1	Surface plasmon resonance	15862300
P59595	NCAP	P62937	PPIA	Surface plasmon resonance	15688292
P59595	NCAP	P63279	UBC9	Other Methods	16998888
P59595	NCAP	P63279	UBC9	Other Methods	17037517
P59595	NCAP	P09651	ROA1	Other Methods	15862300
A7J8L3	A7J8L3	A2A3R6	A2A3R6	Anti tag coimmunoprecipitation	19838190
A7J8L2	A7J8L2	O75348	VATG1	Anti bait coimmunoprecipitation	16226257
A7J8L2	A7J8L2	O75348	VATG1	Anti tag coimmunoprecipitation	16226257
A7J8L2	A7J8L2	O75348	VATG1	Experimental interaction detection	16226257
A7J8L2	A7J8L2	O75348	VATG1	Phage display	16226257
A7J8L2	A7J8L2	O75348	VATG1	ELISA	16226257

LUMIER, luminescence-based mammalian interactome mapping; ELISA, enzyme-linked immunosorbent assay.

**Table 2.** Topological analysis of the human proteins and of the human proteins targeted by SARS-CoV in the human interactome. Cumulative degree and betweenness centrality distributions.

Sl.No	Human Uniprot ID	Human protein	Degree	Betweenness centrality
1	A2A3R6	A2A3R6	1	0.0
2	Q9BYF1	ACE2	4	2271.0
3	Q9H4F8	SMOC1	4	115.53
4	Q9BQB6	VKOR1	8	4431.9
5	P49069	CAMLG	11	3788.8
6	P05155	IC1	13	8518.7
7	O75348	VATG1	15	17898.0
8	Q16548	B2LA1	15	10821.0
9	Q92994	TF3B	19	7621.0
10	Q92843	B2CL2	20	12060.0
11	P08708	RS17	24	15475.0
12	Q07820	MCL1	35	23003.0
13	Q13561	DCTN2	44	32832.0
14	P27448	MARK3	49	32544.0
15	P62937	PPIA	57	90417.0
16	P25787	PSA2	68	26732.0
17	Q07817	B2CL1	84	113360.0
18	P10415	BCL2	97	171060.0
19	O43765	SGTA	133	545880.0
20	O14920	IKKB	134	99858.0
21	P52292	IMA1	143	310540.0
22	P63165	SUMO1	164	334120.0
23	P60866	RS20	170	37806.0
24	P09651	ROA1	174	176900.0
25	P02768	ALBU	178	735080.0
26	P46379	BAG6	205	684320.0
27	P62753	RS6	238	67566.0
28	P63279	UBC9	252	817850.0

**Figure 3.**
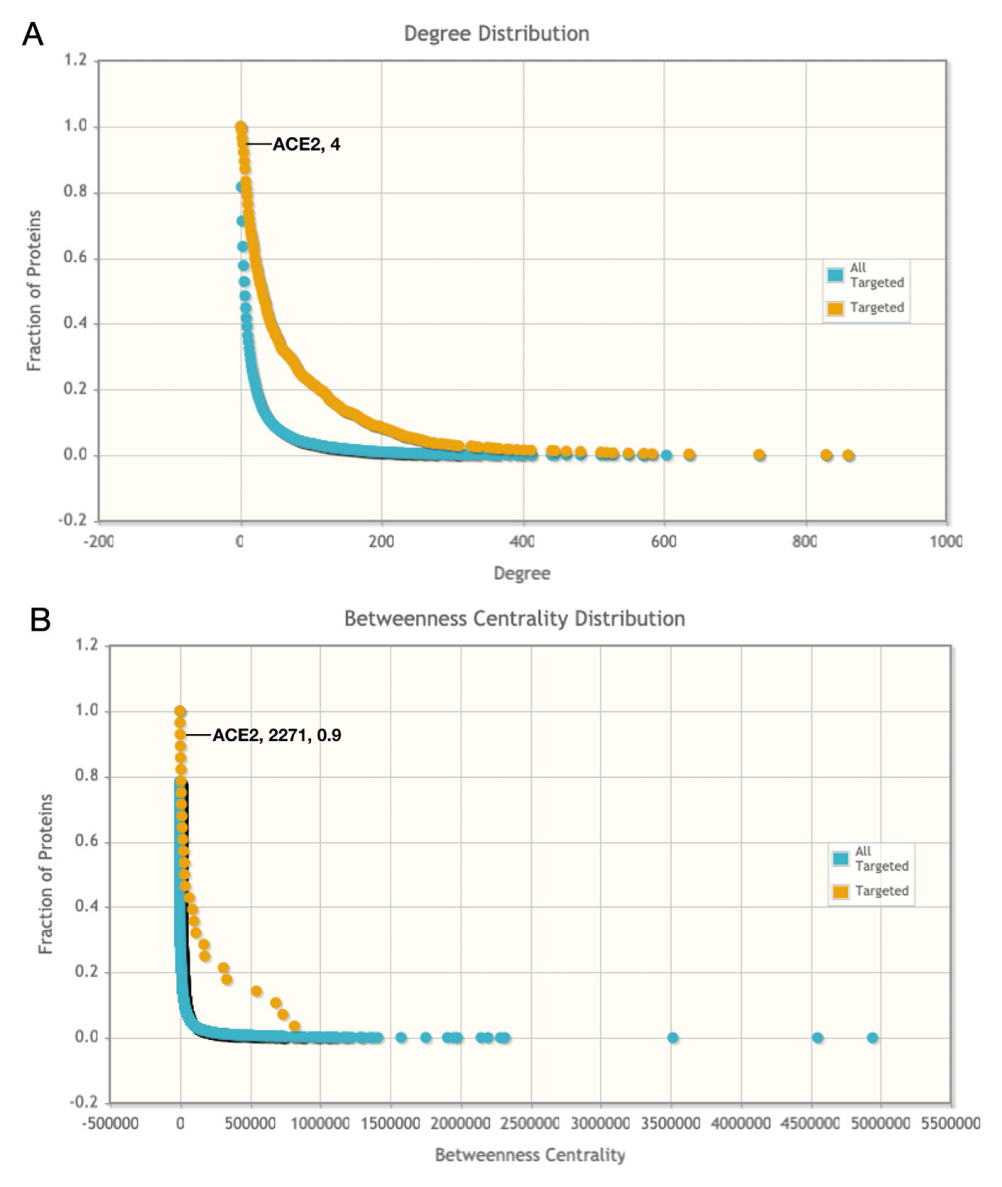
Cumulative degree and betweenness centrality distributions among host proteins involved in the SARS-CoV interaction. Host proteins that are targeted by SARS-CoV have an approximate degree and betweenness centrality. **A**) Degree distribution highlighting ACE2 position and **B**) betweenness centrality distribution highlighting ACE2 position. These findings are statistically significant by the Fligner-Killeen (median) test. This figure was generated using Pathogen-Host Interaction Search Tool (PHISTO).

The degree of connectivity estimates the number of directly connecting neighbours to a particular node, while betweenness centrality estimates the frequency of nodes occurring on the shortest paths in the context of other nodes. In the protein interactomes, a node with a high degree of connectivity is identified as hub protein and a node with maximum betweenness centrality is identified as bottleneck protein. In the current topological analysis, the node with the lowest degree of distribution 1 and betweenness centrality 0.0 was A2A3R6. However, the molecular function of A2A3R6 (Uniprot ID: A2A3R6) is not well understood in both human physiology or pathology. Hence, ACE2, with the degree of distribution 4 and betweenness centrality 2271, was the next most significant node, as shown in Figure 3, and it was identified as a key node or key player in the SARS-CoV/host interaction. Hence, the SARS-CoV-S interaction with host ACE2 was identified as a potential drug target. As information about the SARS-CoV-2/human interaction is not available, the SARS-CoV/human interaction data was used. We studied the similarity between SARS-CoV and SARS-CoV-2 by sequence analysis and RBD prediction.

### Sequence analysis and RBD predictions

**Figure 4.**
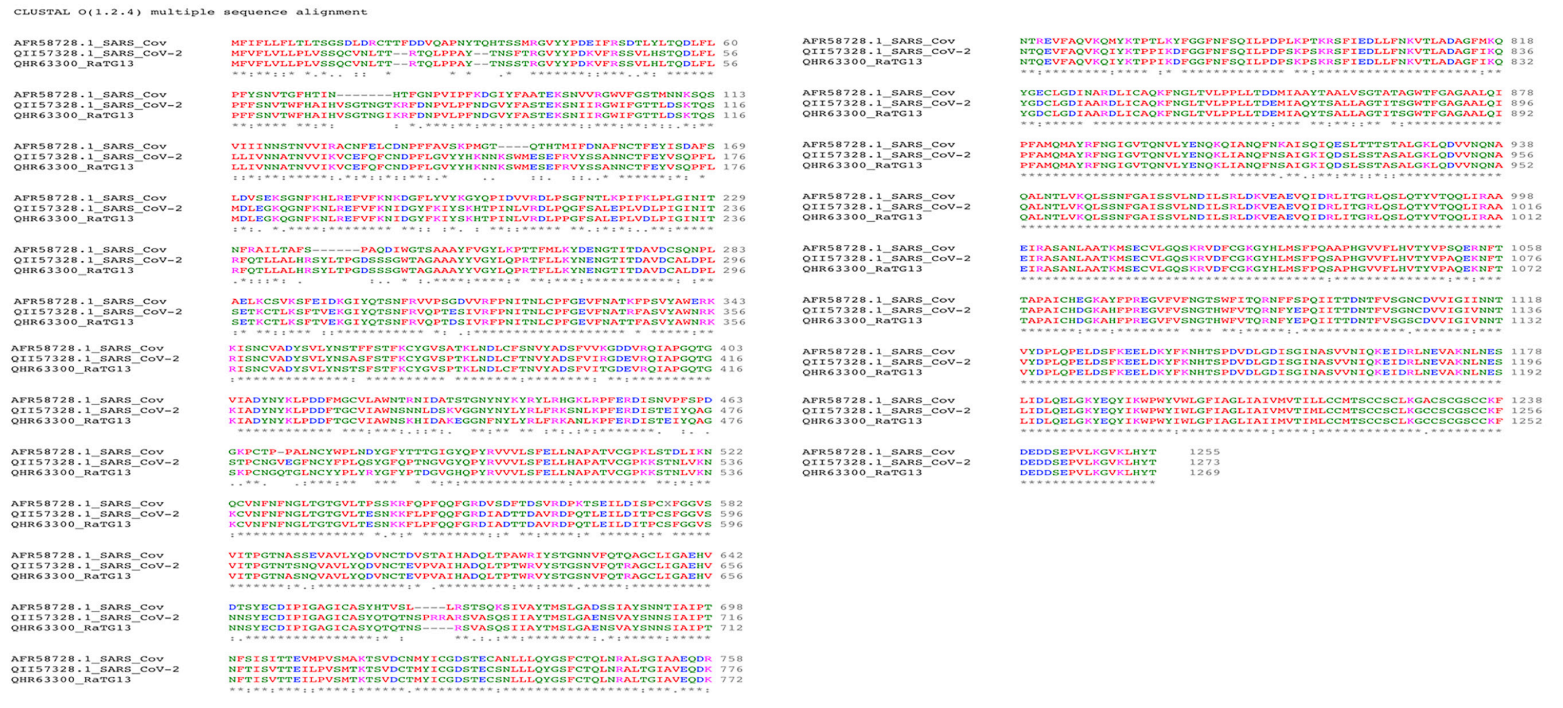
Multiple sequence alignment of S-protein from SARS_CoV-2, SARS-CoV and RatG13 to understand the conservation of amino acids as generated using EMBL-EBI Clustal Omega.

**Figure 5.**
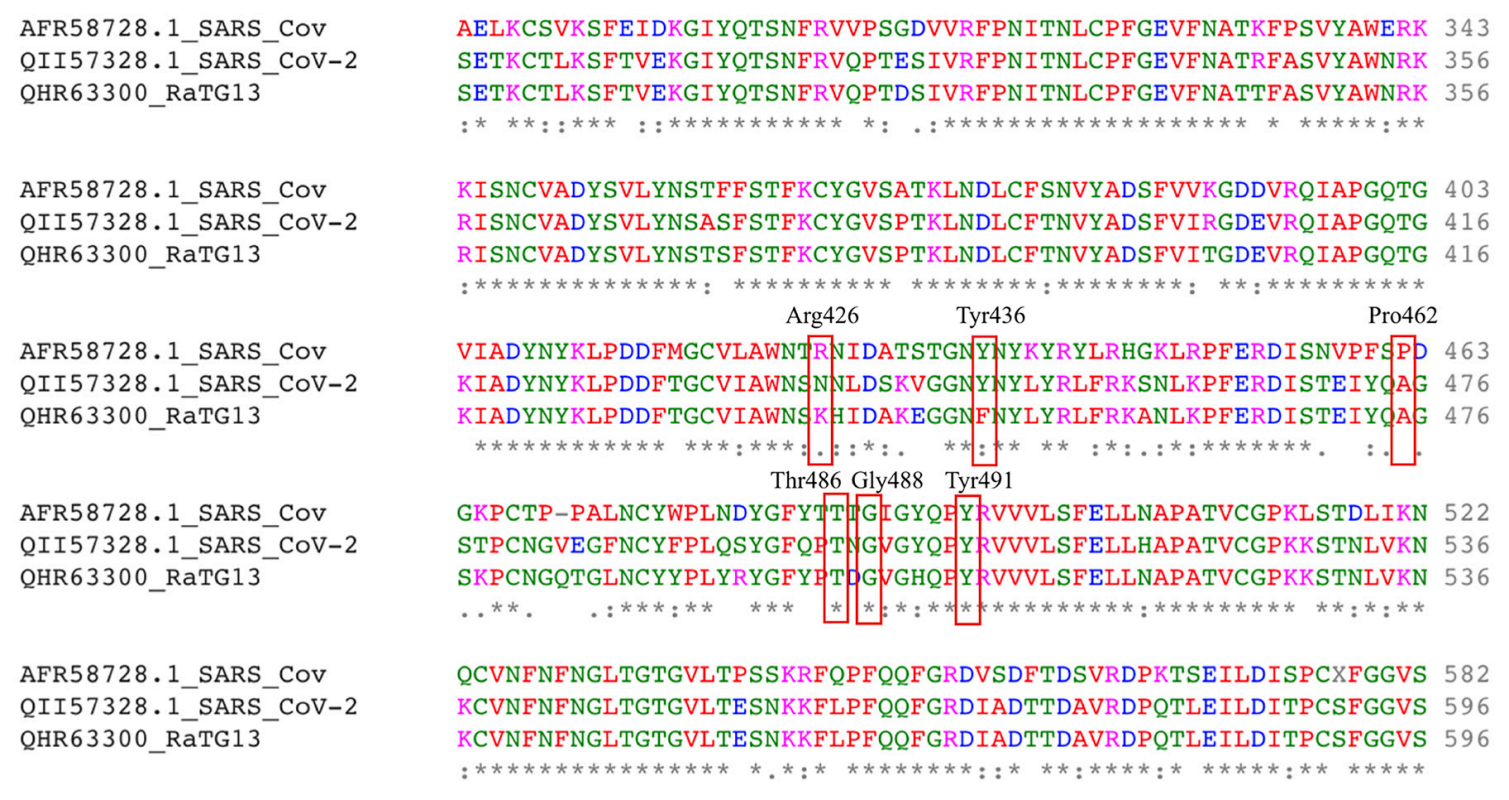
Aligned region of S-protein from SARS_CoV-2, SARS-CoV and RatG13 to understand the conservation of amino acids at the receptor-binding domain region as generated using EMBL-EBI Clustal Omega.

As depicted in Figure 4, the alignment between the S-protein of SARS-CoV-2 and that of Bat coronavirus RaTG13 was closer than with the S-protein of SARS-CoV. The alignment at RBD site residues 317 to 569 was found to be more than 80% similar to SARS-CoV and RaTG13, particularly at major residues including Tyr436, Thr486, Gly488 and Tyr491 but excluding Arg426 and Pro462, as shown in Figure 5. Considering the evolution, the available elucidated structure of the SARS-CoV/ACE2 complex (PDB ID: 6CS2) was used as a template for homology modelling.

**Figure 6.**
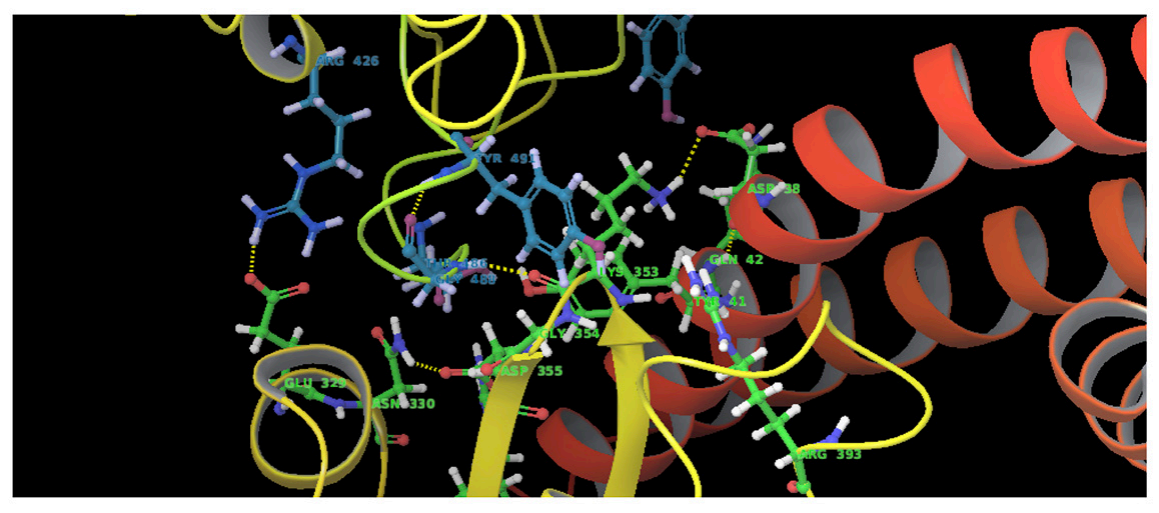
Amino acids involved in the SARS-CoV B-chain and ACE2 D-chain interaction. The residues highlighted in blue correspond to the B-Chain of SARS-CoV and residues in green correspond to the ACE2 D-Chain. This figure was generated using Schrodinger’s Maestro visualizer. As an alternative, the open source visualizer Python Molecule Viewer (PMV) ^48^ could also be used.

The residues involved in the interaction of SARS-CoV with ACE2 were predicted using the Prime module available in Schrodinger Small Molecule Suite and the major interactions are tabulated in Table 3 and shown in Figure 6. A very strong interaction was seen between the smallest amino acid, Gly488, with Lys353, Gly354, and Asp355. This interaction is facilitated by two features; one hydrogen bond and 94.9% buried solvent accessible surface area. Remaining residues also showed significantly strong interactions with ACE2. Hence, the same residues were made centric to generate the grid.

**Table 3.** List of residues involved in SARS-CoV-ACE2 interaction. The residues with hydrogen bonding, better surface complementarity and buried SASA are tabulated and a few other weak interactions are ignored.

Residue	Closest	Distance	Interactions	# HB	# SB	# vWC	SC	B_SASA
**B:426:Arg**	D:329:Glu	2.7 A	2x hb, 1x salt bridge, 1x clash to D:329:Glu	2	1	1	0.43	72.0%
**B:436:Tyr**	D:38:Asp D:42:Gln	3.2 A 3.2 A	1x hb to D:38:Asp	1	0	0	0.75	36.9%
**B:462:Pro**	D:19:Ser D:24:Gln	2.8 A 3.7 A	1x hb to D:19:Ser	1	0	0	0.73	87.3%
**B:486:Thr**	D:41:Tyr D:330:Asn D:355:Asp D:357:Arg	2.8 A 2.9 A 3.1 A 3.8 A	1x hb to D:41:Tyr	1	0	0	0.87	79.8%
**B:488:Gly**	D:353:Lys D:354:Gly D:355:Asp	3.0 A 3.9 A 4.0 A	1x hb to D:353:Lys	1	0	0	0.65	94.9%
**B:491:Tyr**	D:353:Lys D:354:Gly D:393:Arg	3.2 A 3.6 A 3.7 A		0	0	0	0.85	70.0%

HB, hydrogen bond; DS, disulfides, vWC, van der Wsals clash; SC, surface complementarity; B_SASA, buried solvent accessible surface area (SASA).

### Homology modelling and validation of SARS-CoV-2-S protein

**Figure 7.**
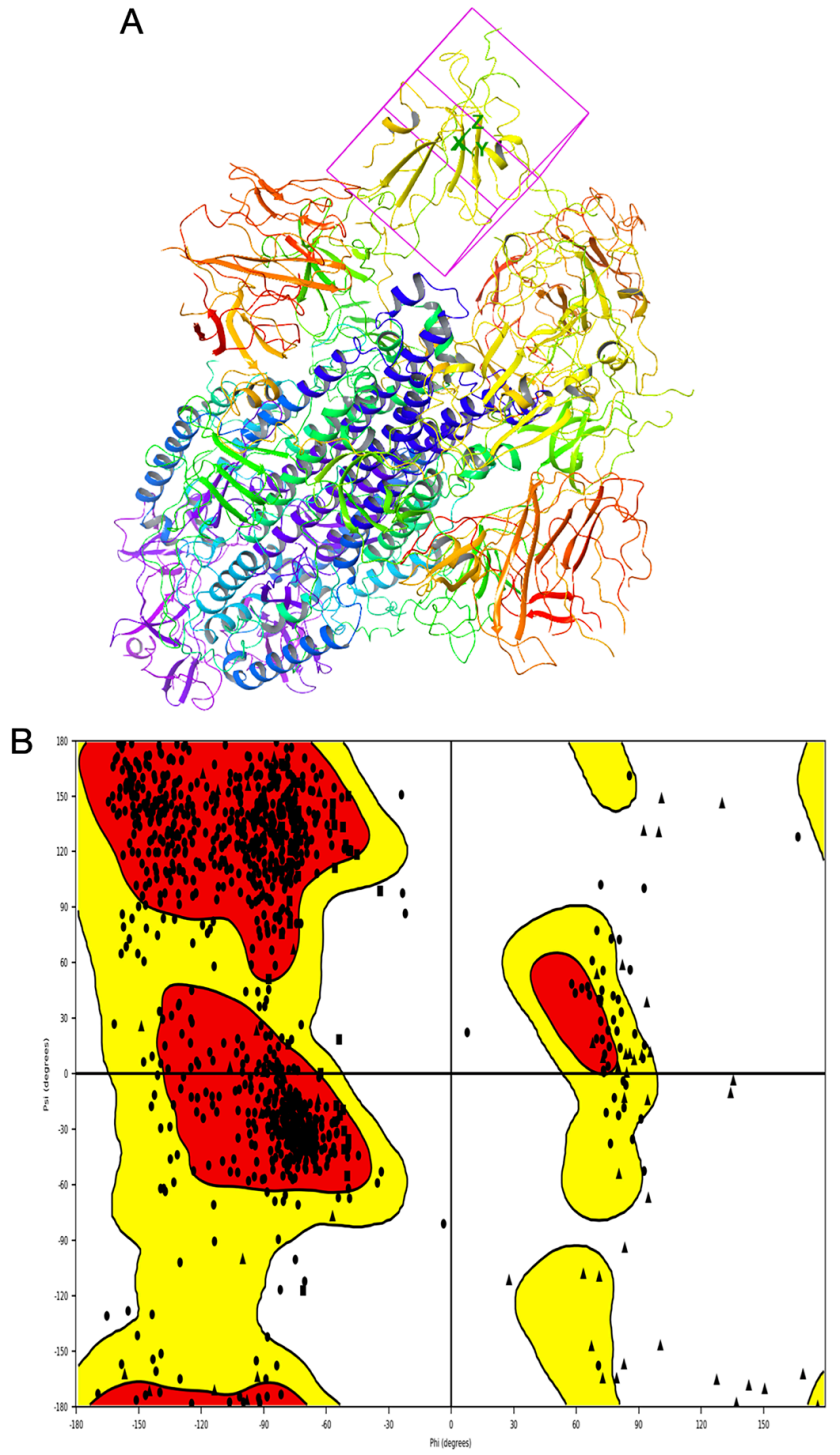
**A**) The modelled structure of SARS-CoV-2 with Arg426, Tyr436, Pro462, Thr486, Gly488 and Tyr491 centric grid box generated for molecular docking, **B**) Ramachandran plot has >95% of amino acids plotted in the allowed region (red and yellow). This figure was generated using Schrodinger’s Maestro visualizer. As an alternative, the open source visualizer Python Molecule Viewer (PMV) ^48^ could also be used.

 The modelling of SARS-CoV-2 was performed using the crystal structure of SARS-CoV-S as a template, which was 97% identical to the query. The modelled protein shown in Figure 7A was validated for quality and preparedness. The Ramachandran plot generated using the protein preparation wizard confirmed the quality of modelled structure by plotting >95% residues in the allowed region, as shown in Figure 7B.

### Molecular docking of small molecules with SARS-CoV-2-S

**Figure 8.**
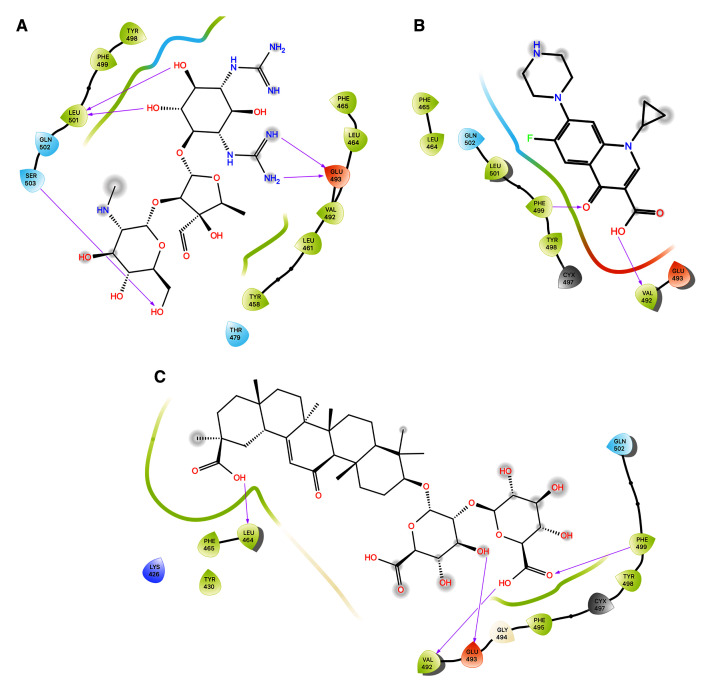
Two-dimensional (2D) illustration of the small molecule interaction in the ACE2 binding site of the receptor-binding domain of SARS-CoV-2-S. **A**) Interaction of streptomycin facilitated by five H-bonds, shown as pink arrows. **B**) Interaction of ciprofloxicine facilitated by two H-bonds, shown as pink arrows. **C**) Interaction of glycyrrhizic acid, facilitated by give H-bonds, shown as pink arrows. This figure was generated using Schrodinger’s Maestro visualizer. As an alternative, the open source visualizer Python Molecule Viewer (PMV) ^48^ could also be used.

The repurposing of small molecules as therapeutics to treat COVID-19 requires knowledge of the interaction of the therapeutic molecule with SARS-CoV-2-S. Initial high-throughput virtual screening suggested 142 molecules that exhibit reasonable interaction with SARS-CoV-2-S. Following this, the shortlisted molecules were docked in SP mode where the accuracy of prediction was improved. The docking in SP mode suggested 15 top molecules, listed in Table 4 as lead molecules. As hydroxychloroquine has been identified as a possible treatment for COVID-19, it was also subjected to subsequent docking in XP mode. All 15 molecules showed better interaction than hydroxychloroquine with SARS-CoV-2-S. The three molecules streptomycin, ciprofloxacin, and GA had low interaction penalties and displayed better interactions with the ACE2 binding site on the RBD of SARS-CoV-2-S, as shown in Figure 8A–C, respectively. The three molecules were selected based on their reported anti-viral activity, safety, availability, and affordability ^45– 47^.

**Table 4.** List of top 15 lead molecules and hydroxychloroquine subjected for molecular docking in XP mode.

Sl. No	Molecule name	XP_Score	Mol_Structure	Pharmacological activity
1	Fytic acid	-10.296	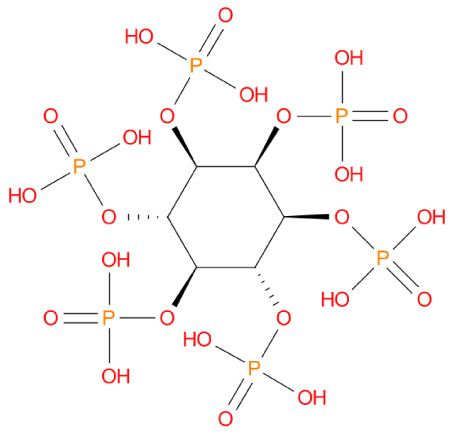	Hypocalcemic agent
2	Diquafosol	-9.109	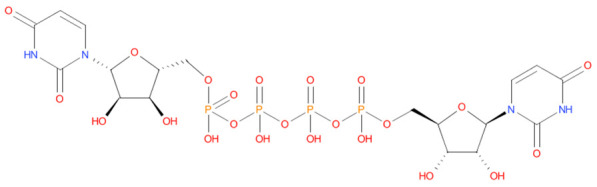	Purinoceptor P2Y(2) receptor agonist
3	Oxiglutatione	-7.869	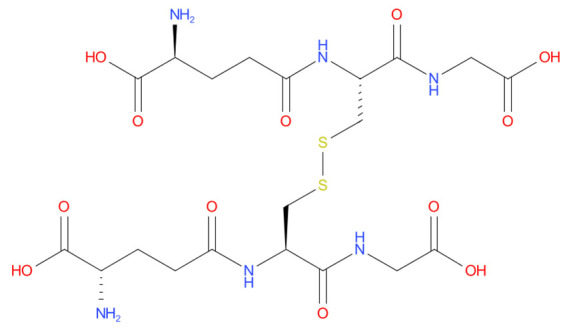	Intraocular Irrigating Solution
4	Ceftolozane	-8.07	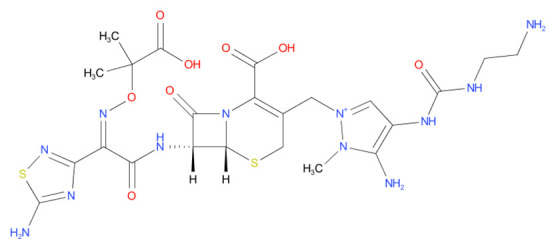	Antibiotic
5	Glycyrrhizic acid	-7.474	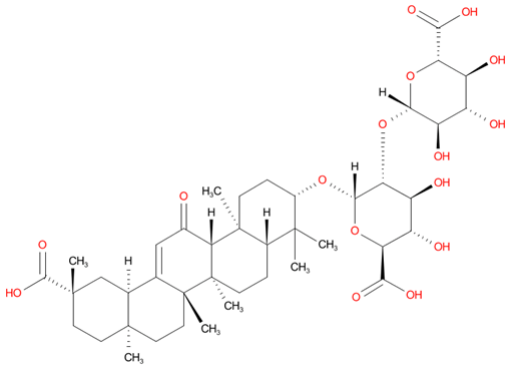	Anti-inflammatory
6	Leucovorin	-7.39	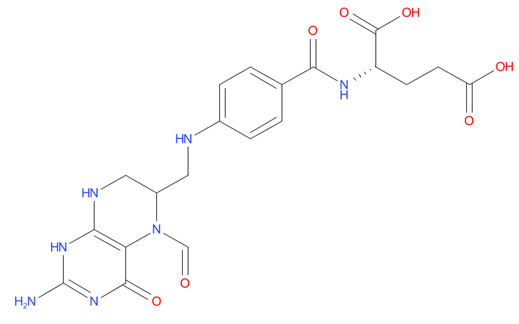	Folic acid antagonist
7	Cefpimizole	-6.51	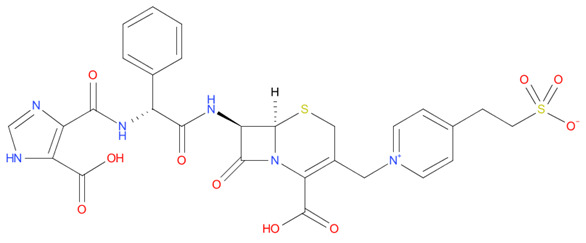	Antibiotic
8	Streptomycin	-6.509	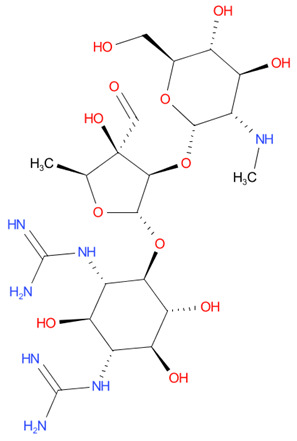	Antibiotic
9	Ertapenem	-5.758	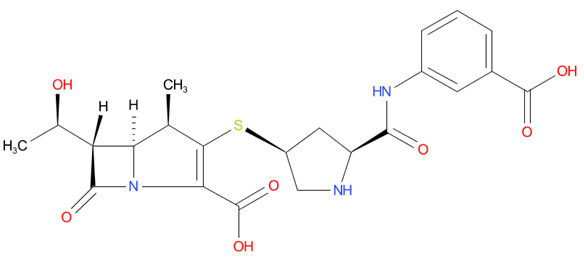	Antibiotic
10	Enoxacin	-5.539	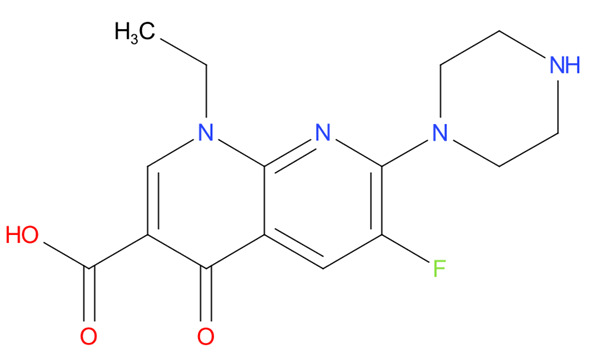	Antibacterial
11	Acetyldigitoxin	-5.407	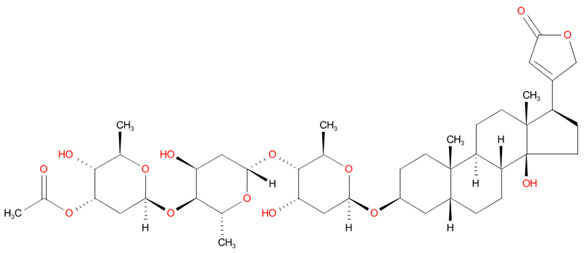	Cardioactive
12	Pefloxacin	-5.399	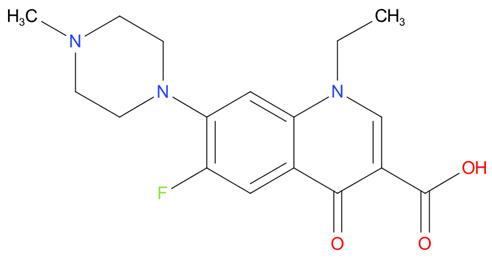	Antibacterial
13	Faropenem	-5.354	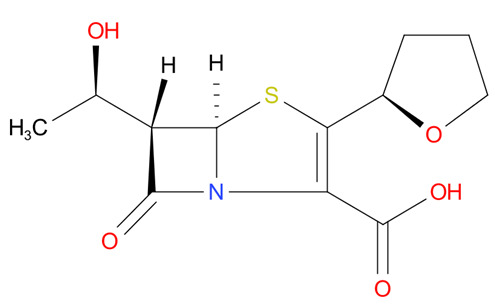	Antibiotic
14	Ciprofloxacin	-5.318	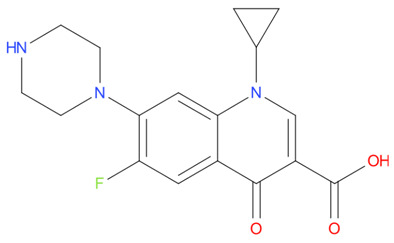	Antibacterial
15	Ceforanide	-5.302	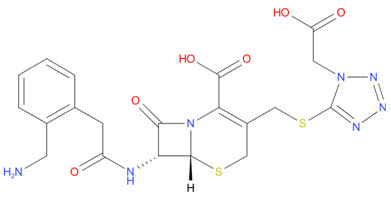	Antibiotic
16	Hydroxychloroquine	-5.302	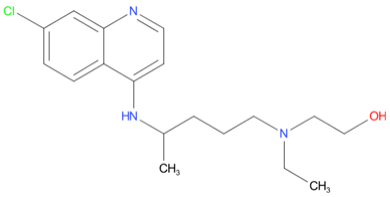	Antimalerial

**Figure 9.**
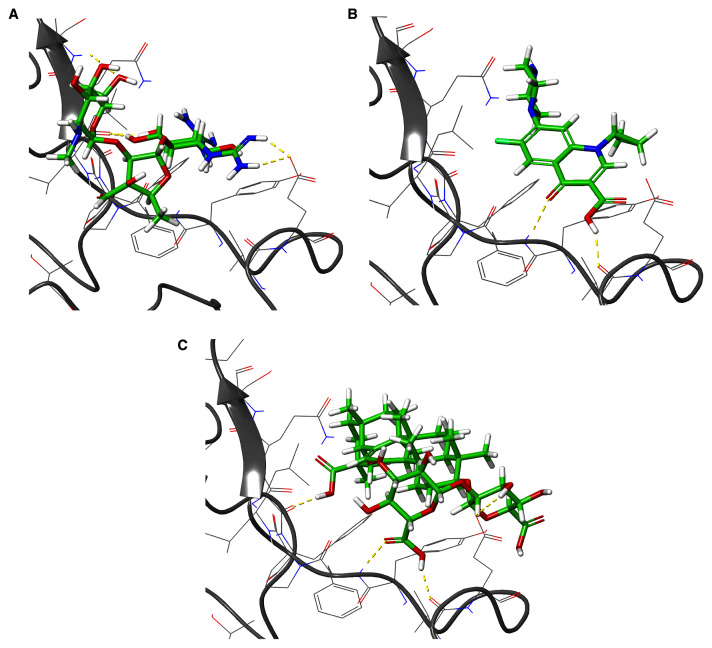
Three-dimensional (3D) illustration of the small molecule interaction in the ACE2 binding site of the receptor-binding domain of SARS-CoV-2-S. **A**) Interaction of streptomycin facilitated by five H-bonds. **B**) Interaction of ciprofloxicine facilitated by two H-bonds. **C**) Interaction of glycyrrhizic acid facilitated by five H-bonds. This figure was generated using Schrodinger’s Maestro visualizer. As an alternative, the open source visualizer Python Molecule Viewer (PMV) ^48^ could also be used.

For SARS-CoV-2-S, the Glide generated docking model showed that streptomycin could bind to SARS-CoV-2-S in a manner highly similar to the SARS-CoV-2-S and ACE2 interaction. The binding pocket of streptomycin was in the RBD site, which has been observed to be an acceptor for ACE2. Streptomycin was well-fitted with the shape of the pocket, as shown in Figure 8A and Figure 9A, with an XP score of -6.5, where it formed a total five hydrogen bonds, among which two hydrogen bonds were formed by donating electrons from N31 and N32 atoms to the Glu493 side-chain atoms. Simultaneously, two other hydrogen bonds were observed between the backbone atoms of Leu501 by receiving electrons from hydroxyl groups at the 5th and 6th carbon atoms of the S1 six-carbon ring of streptomycin. The remaining H-bonds were formed between the backbone atom of Ser503 and the hydroxyl group at the 6th carbon atom at the G3 group of streptomycin. However, the stability of the interaction cannot be pronounced without molecular dynamic simulations.

The docking model of ciprofloxacin illustrated its binding mode on the RBD site, which has been observed to be a key interference site for virus-host interaction. The ciprofloxacin fit with reasonable steric complementarity into the RBD pocket, as shown in Figure 8B and Figure 9B, with an XP score of -5.31. The interaction of ciprofloxacin with SARS-CoV-2-S was facilitated by two hydrogen bonds between Val492 and Phe499; each bond being formed by receiving and donating electrons from hydroxyl and ketone groups, respectively.

The docking model of GA illustrated its binding mode on the RBD site, which has been observed to be a key site for interference of the virus-host interaction. The GA fit with steric complementarity in the RBD pocket, as shown in Figure 8C and Figure 9C, with an XP score of -7.474. The docking of GA with SARS-CoV-2-S was facilitated by three hydrogen bonds with Leu464, Val492, and Glu493 by receiving electrons from the hydroxyl groups of GA. Additionally, the ketone group of GA formed a hydrogen bond, with the backbone atoms of Phe499 receiving the electrons.

### Molecular dynamics simulation of protein-ligand complex

As the SARS-CoV-2-S receptor has 1273aa, it requires enormous computational time to perform MD simulation for the whole range of protein, hence, we confined this study only to the RBD portion, ranging from 317th residue to 569th residue, for MD Simulation.

**Figure 10.**
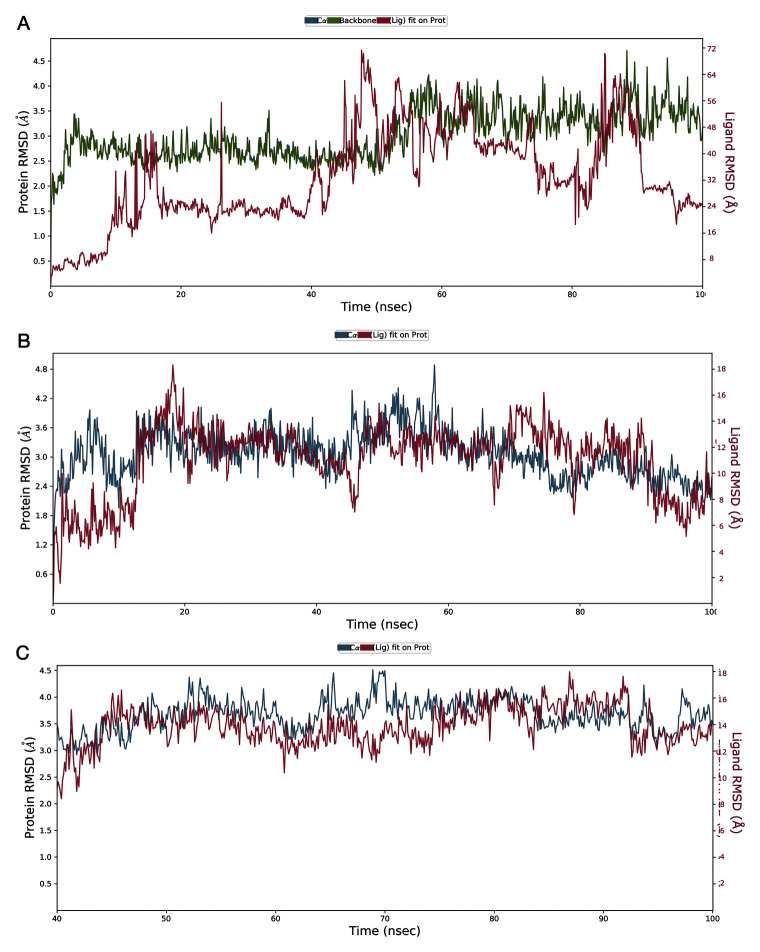
The root mean square deviation (RMSD) plot for protein ligand complexes generated after 100 ns molecular dynamics simulation in the NPT ensemble. **A**) streptomycin/SARS-CoV-2-S complex; **B**) ciprofloxacin SARS-CoV-2-S complex; **C**) glycyrrhizic acid/SARS-CoV-2-S complex. This figure was generated using Desmond version 4.2 ^42^. This analysis could also have been performed using open source software such as GROMACS ^43^.

***RMSD analysis of protein-ligand complex.*** The RMSD can illustrate the average difference in the displacement of selected atoms in a particular frame compared to its reference frame. The plots in Figure 10 illustrate the evolution of a protein (left Y-axis) and ligand (right Y-axis) RMSD. Post simulation, the protein and ligand frames are initially aligned over the backbone atom coordinates of the reference frame, and then the RMSD is extrapolated. The information on protein-ligand RMSD can dissect and demonstrate the conformational differences that occurred throughout the simulation. The RMSD of between 1–3 Å is fairly acceptable for small, globular proteins. An RMSD exceeding this indicates a major conformational change during the simulation and pronounces the instability of the complex.

 The RMSD plot for the streptomycin/SARS-CoV-2-S complex, shown in Figure 10A, attained equilibrium at 5ns and thereafter showed stability with a maximum RMSD of 1 Å (peaks of 2.5 Å - 3.0 Å) up to 55ns. After 55ns a change in the equilibrium state was observed. However, the RMSD was within 1.5 Å, which is acceptable. Similarly, the streptomycin RMSD (right Y-axis) was observed to be significantly higher than the RMSD of the receptor at the RBD site. Thus, it is likely that streptomycin diffuses from its initial binding site after 48ns.

The RMSD plot for the ciprofloxacin/SARS-CoV-2-S complex, shown in Figure 10B, attained equilibrium at 2ns and thereafter showed stability with a maximum RMSD of 1.8 Å (peaks of 2.4 Å - 4.2 Å) up to 58ns. After 58ns a sudden change in equilibrium state was observed. However, the RMSD was within 2 Å, which is acceptable. On the other hand, the RMSD values for ciprofloxacin were observed to be significantly in alignment with the RMSD of SARS-CoV-2-S at the RBD site. Thus, it is likely that ciprofloxacin can retain its initial binding site up to 100ns.

The RMSD plot for the GA/SARS-CoV-2-S complex, shown in Figure 10C, attained equilibrium until 100ns. Compared to SARS-CoV-2-S complexes with streptomycin and ciprofloxacin, it was found that the SARS-CoV-2-S complex with GA was stable until the end of the simulation without any drift in equilibrium. On the other hand, the RMSD values for GA were observed to be significantly in alignment with the RMSD of the SARS-CoV-2-S RBD domain in almost all the frames. Hence, it is likely that it remains in its initial binding site up to 100ns. It is predicted to inhibit SARS-CoV-2-S at the RBD domain comparatively better than streptomycin and ciprofloxacin and for a longer duration, but its contact with key ligands has to be confirmed through RMSF and protein-ligand contact analysis.

**Figure 11.**
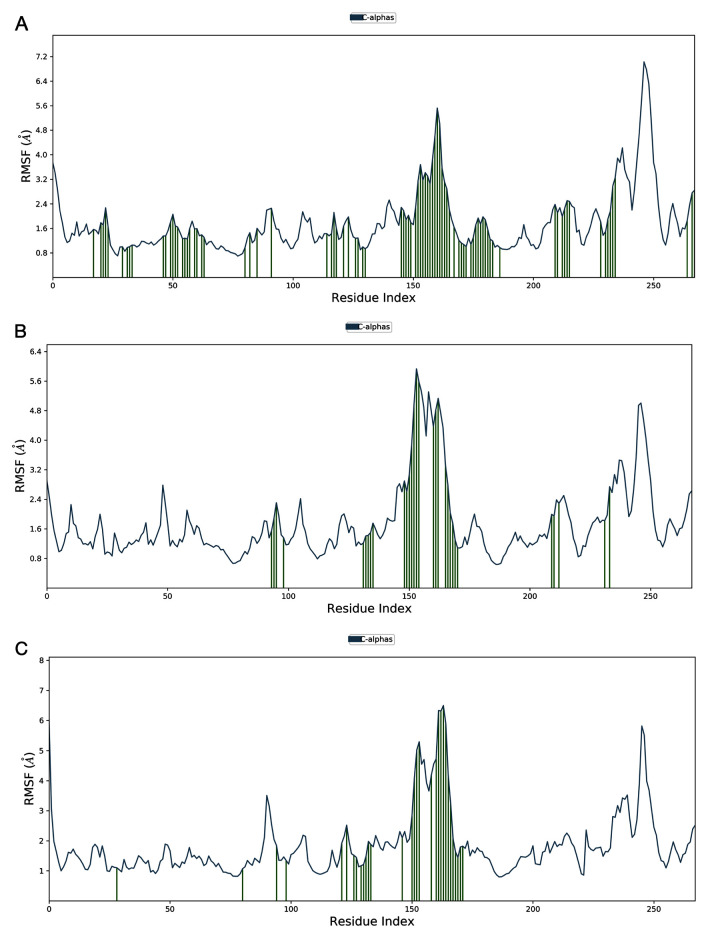
The root mean square fluctuation (RMSF) plot for protein ligand complexes generated after 100 ns molecular dynamics simulation in the NPT ensemble. **A**) streptomycin/SARS-CoV-2-S complex; **B**) ciprofloxacin SARS-CoV-2-S complex; **C**) glycyrrhizic acid/SARS-CoV-2-S complex. Desmond version 4.2 ^42^. This analysis could also have been performed using open source software such as GROMACS ^43^.

***RMSF analysis.*** The RMSF helps characterise minute differences in the protein chain during the simulation. In RMSF plots, peaks correspond to the residues on the protein that fluctuate more during the course of a simulation. Usually, terminals and loop regions fluctuate more than other secondary structures like alpha-helices and beta-strands. The secondary structure of the RBD of SARS-CoV-2-S has the same secondary structural elements as the RBD from SARS-CoV, with 74% homologous residues. These residues are majorly formed of loops and are highly flexible. A unique Phe486 residue in the loop plays a key role in ACE2 interaction by occupying a deep hydrophobic pocket in ACE2. In the trimmed RBD structure this loop starts from 148th residue and ends at 172nd residue. Since the ligand-binding site is located in this loop region, a higher RMSD was noticed. In the RMSF plot for the RBD domain of the streptomycin/SARS-CoV-2-S complex, shown in Figure 11A, the RMSF at the loop region was 5.6Å with many ligand contacts (green-coloured vertical bars). This was on par with molecular docking interactions. In the RMSF plot for the RBD domain of the ciprofloxacin/SARS-CoV-2-S complex, shown in Figure 11B, the RMSF at the loop region was 5.6Å with a few ligand contacts (green-coloured vertical bars). This justifies the interactions seen in molecular docking. Further, in the RMSF plot for the RBD domain of the GA/SARS-CoV-2-S complex, shown in Figure 11C, the RMSF at loop region was 6.3Å with a high number of ligand contacts (green-coloured vertical bars), justifying the interactions seen in molecular docking. Though the ligand contacts are seen in interactions, the simulation time coverage determines their stability.

**Figure 12.**
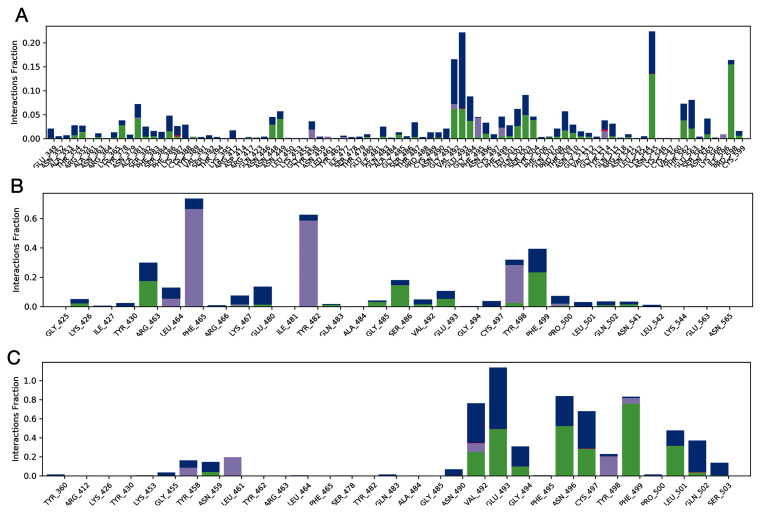
Histogram of protein ligand contacts generated for residues that occur for more than 64.0% of the simulation time in the selected trajectory. **A**) streptomycin/SARS-CoV-2-S complex; **B**) ciprofloxacin SARS-CoV-2-S complex; **C**) glycyrrhizic acid/SARS-CoV-2-S complex. Desmond version 4.2 ^42^. This analysis could also have been performed using open source software such as GROMACS ^43^.

***Protein-ligand contacts.*** Protein-ligand interactions can be traced throughout the simulation and can be categorised into four types: hydrogen bonds, hydrophobic, ionic, and water bridges, as summarised in Figure 12A–12C. The stacked bars in the plots are normalised over the course of the trajectory and help us to understand the retention of contact throughout the simulation time. The contacts with a value of more than 0.7 are expected to be retained for over 70% of the total simulation time.

In the protein-ligand contact plot for the streptomycin/SARS-CoV-2-S complex, shown in Figure 12A, residues Glu493 and Lys544 showed maximum interactions fractions, i.e. 0.20 facilitated by hydrogen bonds and water bridges. This suggests that the specific interaction is maintained for 20% of the simulation time, and such short interactions are not promising. Hence, streptomycin cannot be a potential inhibitor of SARS-CoV-2-S to offer anti-COVID-19 activity.

In the protein-ligand contact plot for the ciprofloxacin/SARS-CoV-2-S complex shown in Figure 12B, residues Phe465, Tyr482, Tyr498, and Phe499 were seen to have the interactions fractions 0.75, 0.6, 0.35 and 0.39 respectively facilitated by hydrophobic, hydrogen bonds and water bridges. This suggests that for 70%, 60%, 35% and 39% of the simulation time, the specific interaction is maintained by respective residues and such interactions are considered good. Hence, ciprofloxacin may be a potential inhibitor of SARS-CoV-2-S and may offer anti-COVID19 activity.

In the protein-ligand contact plot for the GA/SARS-CoV-2-S complex shown in Figure 12C, residues Val492, Glu493, Asn496, Cys497, and Phe499 had the interactions fractions 0.78, 1.12, 0.80, 0.60 and 0.80, respectively, facilitated by hydrophobic, hydrogen bonds and water bridges. This suggests that for 78%,100%, 80%, 60% and 80% of the simulation time, the specific interaction is maintained by respective residues and such interactions are excellent and promising. Hence, GA can be a potential inhibitor of SARS-CoV-2-S and can offer anti-COVID19 activity.

## Conclusion

Through our topological analysis, we have determined the degree of distribution for viral proteins, and we show that, due to its low degree of distribution, ACE2 is likely to be targeted by viruses like SARS-CoV. Hence, the interaction between the viral protein SARS-CoV-2-S and the host ACE2 receptor is a potential drug target for the repurposing of known drugs. Further, sequence alignment and domain analysis suggest that the RBD is the ligand-binding site. Molecular docking studies have suggested streptomycin, ciprofloxacin, and GA as possible leads to inhibit SARS-CoV-2-S. Molecular dynamic simulation analysis has indicated that GA is a promising small molecule that could be repurposed as a potential inhibitor of SARS-CoV-2-S to offer anti-COVID19 activity.

## Data availability

All data underlying the results are available as part of the article and no additional source data are required.
