# Supplementary material for: In silico screening of known small molecules to bind ACE2 specific RBD on Spike glycoprotein of SARS-CoV-2 for repurposing against COVID-19
Source: F1000Res. 2020 Jul 1;9:663. [Version 1] doi: 10.12688/f1000research.24143.1 (PMC7385708; doi:10.12688/f1000research.24143.1)
Supplement: Peer review history

# Peer review: Reviewer response for version 1

**DOI:** 10.5256/f1000research.26631.r67868

**Published:** 2020-07-27

**Authors:** Raghavendra HL

**Affiliations:** 1 College of Medical and Health Sciences, Wollega University, Nekemte, Ethiopia

The manuscript entitled “ *In silico* screening of known small molecules to bind ACE2 specific RBD on Spike glycoprotein of SARS-CoV-2 for repurposing against COVID-19” addresses the repurposability of known drugs against COVID-19. Spike glycoprotein is the most popular drug target for SARS-CoV-2 having a significant role in host entry. With this basic understanding, the manuscript is reviewed and the observations are listed below.

- Abstract: Conclusion:

 The statement “Hence, the molecules screened for inhibitory properties against SARS-CoV-2-S can be clinically used to treat COVID-19 since the safety profile is already known.” can be rewritten as “The GA of plant origin shown superior interaction with SARS-CoV-2-S compared to rest other molecules, hence, GA can be clinically investigated to confirm its efficacy to treat COVID-19.”

- Methods: Homology modelling of SARS-CoV-2-S protein

The crystal structure of SARS-CoV-S and ACE2 complex (PDB ID: 6ACD) is used as a template for homology modelling. It is expected to discuss the identity, query coverage between the query and template. Discuss the criteria for template selection.

- In the figure legend for Figures 8c, 9c, 10c, 11c, and 12c glycyrrhizic acid can be replaced by GA.

The manuscript can be accepted for indexing with above-mentioned changes.

Is the work clearly and accurately presented and does it cite the current literature?

Yes

If applicable, is the statistical analysis and its interpretation appropriate?

Yes

Are all the source data underlying the results available to ensure full reproducibility?

Yes

Is the study design appropriate and is the work technically sound?

Yes

Are the conclusions drawn adequately supported by the results?

Yes

Are sufficient details of methods and analysis provided to allow replication by others?

Yes

Reviewer Expertise:

Gestational Diabetes Mellitus, Omics, Drug Discovery, Phytopharmacology and Bioinformatics

I confirm that I have read this submission and believe that I have an appropriate level of expertise to confirm that it is of an acceptable scientific standard.

## References

[1] : In silico screening of known small molecules to bind ACE2 specific RBD on Spike glycoprotein of SARS-CoV-2 for repurposing against COVID-19. *F1000Research*.2020;9: 10.12688/f1000research.24143.1 10.12688/f1000research.24143.1 PMC738570832765844

# Peer review: Reviewer response for version 1

**DOI:** 10.5256/f1000research.26631.r66947

**Published:** 2020-07-14

**Authors:** S.M. Vidya

**Affiliations:** 1 Department of Biotechnology, NMAM Institute of Technology, Udupi, Karnataka, India

In the present study authors have made an effort to repurpose the known drugs against SARS-CoV-2. Although the study is well organized and presented, a few minor changes are suggested for betterment. 

1. The name of the molecules should begin with Uppercase.
2. In an abstract, authors have mentioned about the  anti-viral activity of streptomycin, ciprofloxacin, and glycyrrhizic acid. But, it requires the literature support. Authors can cite the references for the anti-viral/ ant-microbial activity in Methods section.
3. In Results and Discussions, authors could claim the better affinity of glycyrrhizic acid towards SARS-CoV-2-S in comparison with other two molecules. Change suggested: Additionally, the ketone group of GA formed a hydrogen bond, with the backbone atoms of Phe499 receiving the electrons and possess the better affinity towards SARS-CoV-2-S when compared to Streptomycin and Ciprofloxicine.
4. In conclusion section, authors have mentioned that, ACE2 is likely to be targeted by viruses like SARS-CoV. Since, authors have tabulated the methods and references for ACE2 and SARS-CoV spike protein interaction in table 1, it is better to rewrite as "ACE2 is known to be targeted by viruses like SARS-CoV".

Is the work clearly and accurately presented and does it cite the current literature?

Yes

If applicable, is the statistical analysis and its interpretation appropriate?

Not applicable

Are all the source data underlying the results available to ensure full reproducibility?

Yes

Is the study design appropriate and is the work technically sound?

Yes

Are the conclusions drawn adequately supported by the results?

Yes

Are sufficient details of methods and analysis provided to allow replication by others?

Yes

Reviewer Expertise:

Phytochemical screening, Pharmacology, Molecular docking

I confirm that I have read this submission and believe that I have an appropriate level of expertise to confirm that it is of an acceptable scientific standard.
